# A Pilot Study on Circulating, Cellular, and Tissue Biomarkers in Osteosarcopenic Patients

**DOI:** 10.3390/ijms25115879

**Published:** 2024-05-28

**Authors:** Francesca Salamanna, Cesare Faldini, Francesca Veronesi, Veronica Borsari, Alberto Ruffilli, Marco Manzetti, Giovanni Viroli, Matteo Traversari, Laura Marchese, Milena Fini, Gianluca Giavaresi

**Affiliations:** 1 Surgical Sciences and Technologies, IRCCS Istituto Ortopedico Rizzoli, Via di Barbiano 1/10, 40136 Bologna, Italy; francesca.salamanna@ior.it (F.S.); veronica.borsari@ior.it (V.B.); laura.marchese@ior.it (L.M.); gianluca.giavaresi@ior.it (G.G.); 2 1st Orthopaedic and Traumatologic Clinic, IRCCS Istituto Ortopedico Rizzoli, Via di Barbiano 1/10, 40136 Bologna, Italy; cesare.faldini@ior.it (C.F.); alberto.ruffilli@ior.it (A.R.); marco.manzetti@ior.it (M.M.); giovanni.viroli@ior.it (G.V.); matteo.traversari@ior.it (M.T.); 3 Department of Biomedical and Neuromotor Science (DIBINEM), University of Bologna, Via Zamboni 33, 40126 Bologna, Italy; 4 Scientific Direction, IRCCS Istituto Ortopedico Rizzoli, Via di Barbiano 1/10, 40136 Bologna, Italy; milena.fini@ior.it

**Keywords:** osteosarcopenia, osteopenia, sarcopenia, biomarkers, aging

## Abstract

Aging comes with the loss of muscle and bone mass, leading to a condition known as osteosarcopenia. Circulating, cellular, and tissue biomarkers research for osteosarcopenia is relatively scarce and, currently, no established biomarkers exist. Here we find that osteosarcopenic patients exhibited elevated basophils and TNFα levels, along with decreased aPPT, PT/INR, IL15, alpha-Klotho, DHEA-S, and FGF-2 expression and distinctive bone and muscle tissue micro-architecture and biomarker expressions. They also displayed an increase in osteoclast precursors with a concomitant imbalance towards spontaneous osteoclastogenesis. Similarities were noted with osteopenic and sarcopenic patients, including a lower neutrophil percentage and altered cytokine expression. A linear discriminant analysis (LDA) on models based on selected biomarkers showed a classification accuracy in the range of 61–78%. Collectively, our data provide compelling evidence for novel biomarkers for osteosarcopenia that may hold potential as diagnostic tools to promote healthy aging.

## 1. Introduction

Osteoporosis and sarcopenia are widespread geriatric conditions prevalent in the elderly population [1,2,3,4]. Osteoporosis is characterized by a decrease in bone mineral density (BMD) with age and/or menopause [1], often resulting in osteoporosis fractures [2]. In contrast, sarcopenia is a skeletal muscle disorder marked by the gradual and generalized loss of muscle mass and strength [3,4]. Both geriatric conditions elevate the risks of falls, fractures, disability, and frailty [1,2,3,4]. When osteoporosis and sarcopenia occur together, a condition termed osteosarcopenia can be established [5]. Collectively, these pathological conditions can elevate the risk of adverse outcomes that impact the quality of life and physical independence of patients, potentially leading to disability and premature mortality [5]. The prevalence of osteosarcopenia has been reported to range from 10–15% in community-dwelling older adults [6] to approximately 10% in those attending outpatient frailty clinics [7,8], and even as high as 64.3% in osteoporotic outpatient clinics [8,9,10].

Recent research suggests that the connection between bone and muscle is, in part, driven by a phenomenon known as bone–muscle crosstalk [11]. These tissues are intricately linked in terms of their anatomy, mechanical functions, and biochemical interactions and can communicate through paracrine and endocrine-like signaling mechanisms [11]. Changes in mechanical loading, particularly through activities like resistance exercise, have an intense impact on both muscle and bone [11,12,13]. Increased mechanical loading can lead to muscle growth, while reduced loading can result in muscle mass and strength decline [11]. Furthermore, mechanical loading plays a regulatory role in bone health, allowing it to adapt its mass, shape, and micro-architectural properties in response to varying mechanical stimuli [12,13]. For example, prolonged bed rest [12] and immobilization [13] are known to have detrimental effects on both muscle and bone. Concerning the biochemical interactions between bone and muscle, they are bidirectional and can have systemic effects on distant tissues and organs [11]. Muscles release various secretory factors, including myokines, peptides, growth factors, and hormones, which can influence bone health independently of mechanical loading [14]. Several of these signaling molecules have a local and systemic impact on metabolism, affecting distant organs. Some well-known muscle myokines include myostatin, the leukemia inhibitory factor (LIF), irisin, β-aminoisobutyric acid (BAIBA), insulin-like growth factors (IGF-1), fibroblast-like growth factors (FGF)-2, interleukin (IL)6, IL10, and IL15 [14]. Several of them, such as FGF2, IL6, and IL15 [14], have been implicated in bone–muscle crosstalk, either positively or negatively. It is believed that myokines can impact bone health directly by binding to specific receptors or indirectly through interactions with other tissues, such as adipose tissue [14]. While bones do not release secretory factors in the same way muscles do with myokines, they are dynamic tissues that play a crucial role in the endocrine system. In fact, the concept of the skeleton as an endocrine organ has shifted our understanding away from perceiving bone solely as a support and protection structure. Various factors contribute to bone–muscle crosstalk, including the lacunocanalicular system, osteoclasts, osteoblast and osteocyte factors, and bone marrow mesenchymal cells, as well as numerous mediators, factors, and osteokines secreted by these cells, i.e., osteoprotegerin (OPG), osteocalcin, sclerostin, receptor activator of nuclear factor kappa-Β ligand (RANKL), bone morphogenetic proteins (BMPs), tumor necrosis factor (TNF)-α, and specific non-collagenous and collagenous proteins such as collagen type I alpha 1 chain (COL1A1) and tartrate-resistant acid phosphatase (TRAP) [15]. In addition to myokines, cytokines, and definite bone and muscle mediators, other factors can play a key role in bone–muscle interactions, such as hormones that can potentially influence bone density and muscle mass such as dehydroepiandrosterone (DHEA) and estradiol (E2), and specific longevity-related proteins like alpha-Klotho that are able to influence the secretion of myokines, which in turn are able to play a critical role in the communication with bones [14].

Despite the identification of several factors that contribute to bone–muscle crosstalk, specific biomarkers of osteosarcopenia have not been adequately identified. This study aimed to explore circulating, cellular, and tissue biomarkers in osteosarcopenic (OS) patients comparing them with healthy, osteopenic (OP), and sarcopenic (SP) patients. In detail, we analyzed and evaluated routine blood test parameters, peripheral blood mononuclear cells (PBMCs), spontaneous osteoclastogenesis (the activation of osteoclasts without specific external stimulation or inducement, a risk factor in pathological conditions characterized by altered BMD), bone and muscle micro-architecture, and physiological and pathological expression of blood serum and tissue markers including those for bone, muscle, and aging. In detail, skeletal and muscle myokines such as IL15, CNTF, and FGF-2, mediators of inflammation and pro-inflammatory cytokines such as PGE2 and IL-6, TNF-α, IL-1β, bone-protecting cytokines such as IL10 and IL33/ST2 signaling, proteins linked to aging-like phenotypes such as α-Klotho, and hormones affecting bone and muscle cells such as DHEA-sulfate were evaluated in patients’ sera. Additionally, morphological and histological bone and muscle structure and quality and specific tissue markers of bone turnover and muscle metabolism such as OPG, RANKL, BMP2, TNFα, and COL1A1 were assessed and quantified.

## 2. Results

### 2.1. Demographics Data and Baseline Clinical Characteristics

Baseline clinical characteristics and demographics data are reported in Table 1. Eighteen patients were included in the study, comprising twelve (67%) females and six (33%) males. The mean age at vertebral fusion surgery was 63.9 years (range 44–82 years) and the body mass index (BMI) was 25.19 kg/m^2^ (range 20.8–32.3 kg/m^2^). The average psoas to lumbar vertebral index (PLVI) was 0.83 (range 0.64–1.29) with a mean M-Score of 2.62 (range 0.82–5.09). In our cohort, 50% of patients had PLVI values below the average, while the remaining 50% had values above the average. Regarding the M-Score, 44% of patients had values below the average, and 56% had values above the average. Out of the 18 patients, 5 were diagnosed with osteopenia, 6 with sarcopenia, 4 with osteosarcopenia, and 3 did not exhibit sarcopenia and/or osteopenia, serving as the Control group. The length of stay averaged 12.7 days (range 5–30 days). Finally, no significant differences were found among the groups of patients for all data reported in Table 1.

Regarding hematological parameters, the percentages of neutrophils and basophils, prothrombotic activity (INR), and activated partial thromboplastin time (aPPT) were significantly different among OP, SP, OS, and Control patients and among OP, SP, and OS patients, even though they are all within the normal range. The percentage of neutrophils was significantly lower in OP (*p* = 0.031), SP (*p* = 0.001), and OS (*p* = 0.002) patients in comparison to Control patients (Table 2). The percentage of basophils was significantly higher in OS patients (*p* = 0.006) than in Controls, and those of OP (*p* = 0.030) and SP (*p* = 0.004) were significantly lower compared to OS patients. In addition, INR and aPPT results showed significantly lower values in OS (INR, *p* = 0.003; aPTT, *p* = 0.05) than in Control patients and higher values in OP (INR, *p* = 0.032; aPTT, *p* = 0.040) and SP (INR, *p* = 0.012; aPTT, *p* = 0.024) in comparison to OS patients. All other hematological parameters are given in Supplementary File Table S1.

**Figure 1:**
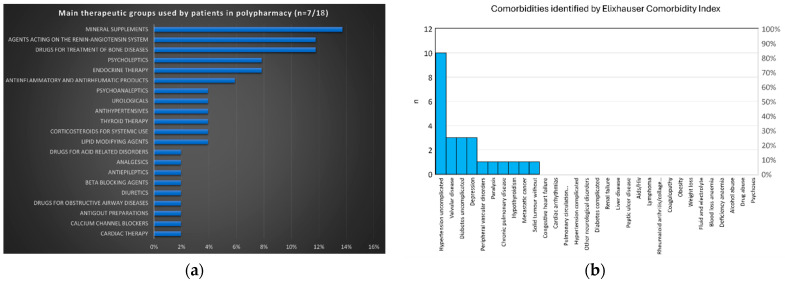
Percentage of drug class administered to patients in polypharmacy (**a**) and comorbidities identified for patients included in the study (**b**).

As reported in Figure 1, the main therapeutic groups used by patients in polypharmacy were mineral supplements, agents acting on the renin–angiotensin system, and drugs used for the treatment of bone disease, while the most frequent comorbidity was uncomplicated hypertension, followed by valvular disease, uncomplicated diabetes, and depression. Table 3 shows the results of the Elixhauser Comorbidity Index (ECI), a method of categorizing comorbidities of patients based on the International Classification of Diseases (ICD) diagnosis codes found in administrative data and polypharmacy; no significant differences were observed among the groups of patients.

### 2.2. Serum Measurement

**Figure 2:**
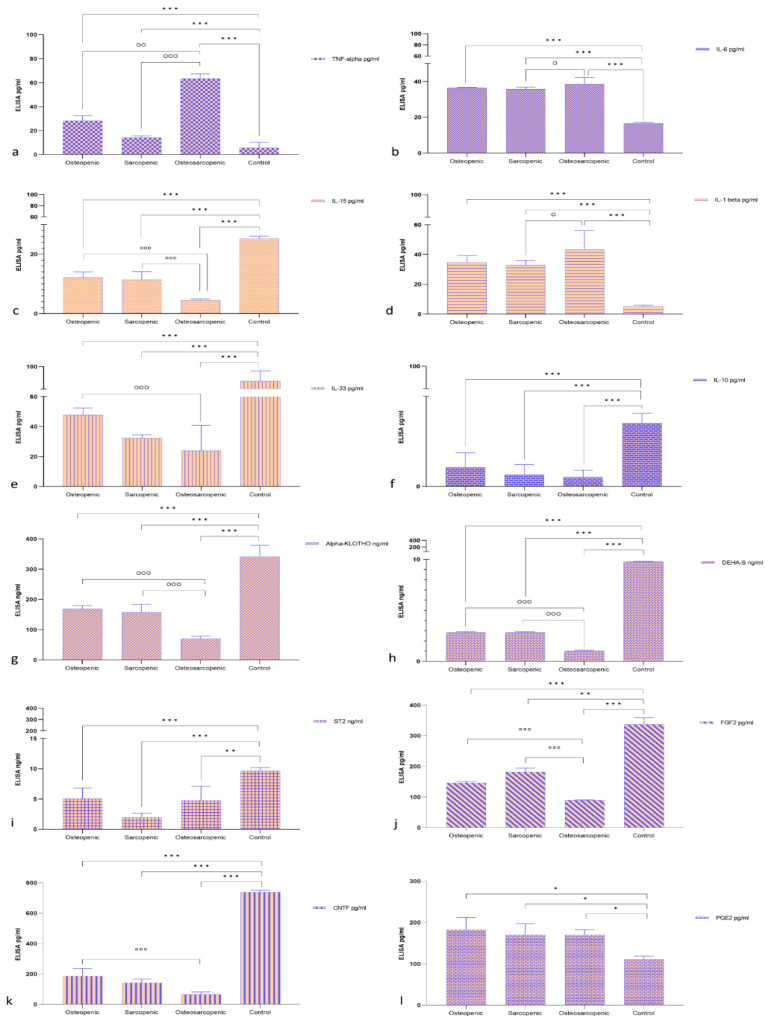
Bar plots of the serum levels of selected markers: (**a**) TNF-α, (**b**) IL-6, (**c**) IL-15, (**d**) IL-1β, (**e**) IL-33, (**f**) IL-10, (**g**) Alpha-KLOTHO, (**h**) DEHA-S, (**i**) ST2, (**j**) FGF2, (**k**) CNTF, and (**l**) PGE2 serum levels (Mean ± SD). One-way ANOVA followed by Dunnett’s test of OP, OS, and SP vs. Control (*) and OP and SP vs. OS (°): 1 symbol, *p* < 0.05, 2 symbols, *p* < 0.005, 3 symbols, *p* < 0.0005.

Sera levels of selected markers linked to bone, muscle, and aging were assessed (Figure 2a–l). All selected serum biomarkers of the OP, SP, and OS groups had significant differences compared to the Control group; specifically, they were lower in IL15, IL33, IL10, Alpha-KLOTHO, DEHA-S, ST2, FGF2, and CTNF and higher in TNF-α, IL-6, IL-1β, and PGE2, respectively. TNF-α showed significantly higher levels in the OS patients compared to the OP (*p* < 0.005) and SP groups (*p* < 0.0005) (Figure 2a). Similarly, serum levels of IL-6 (Figure 2b) and IL-1β (Figure 2d) in OS patients were also significantly (*p* < 0.05) higher than in SP patients. In contrast, serum levels for IL15, Alpha-KLOTHO, DEHA-S, and FGF2 in OS patients were significantly lower than in SP and OP patients. Finally, except for serum levels of IL10 and PGE2, for all remaining biomarkers, the levels observed in OS patients were also significantly (*p* < 0.005) lower than those in OP (IL15, IL33, Alpha-KLOTHO, DEHA-S, FGF2, and CNTF) patients.

### 2.3. Spontaneous Osteoclastogenesis

**Figure 3:**
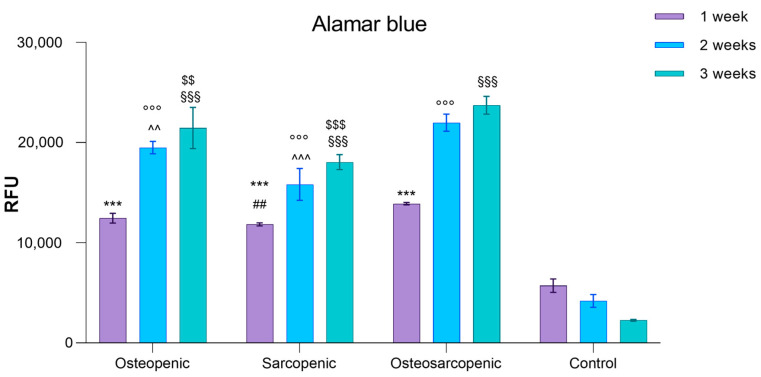
Bar plots of the PBMCs viability of OP, SP, OS, and Control patients after 1, 2, and 3 weeks of culture (Mean ± SD, n = 3 triplicates). Two-way ANOVA (F = 198.8, *p* < 0.0005) followed by Dunnett’s test of OP, OS, and SP vs. Control (*, at 1 week; °, at 2 weeks; and §, at 3 weeks) and OP and SP vs. OS (#, at 1 week; ^, at 2 weeks; and $, at 3 weeks): 2 symbols, *p* < 0.005, 3 symbols, *p* < 0.0005.

Given that osteoclasts are multinucleated bone-resorbing cells that can be differentiated from human peripheral blood mononuclear cells (PBMCs) in vitro, and considering the several studies that have demonstrated an increase in spontaneous osteoclastogenesis (the formation of osteoclasts without the need for differentiating factors required for PBMC viability and differentiation into osteoclasts in healthy conditions) in osteoporotic conditions, we evaluated PBMC viability and spontaneous osteoclastogenesis in all patients. The Alamar Blue test revealed significantly higher viability in PBMCs from OP, SP, and OS patients compared to those of Control patients at each experimental time point (*p* < 0.0005) (Figure 3). Additionally, after one week, OS PBMCs exhibited higher viability compared to SP PBMCs (*p* = 0.004) (Figure 3). After two and three weeks, OS PBMCs showed higher viability compared to both OP and SP PBMCs (2 weeks: OS vs. OP, *p* = 0.001; OS vs. SP, *p* < 0.0005; 3 weeks: OS vs. OP, *p* = 0.002; OS vs. SP, *p* < 0.0005) (Figure 3).

**Figure 4:**
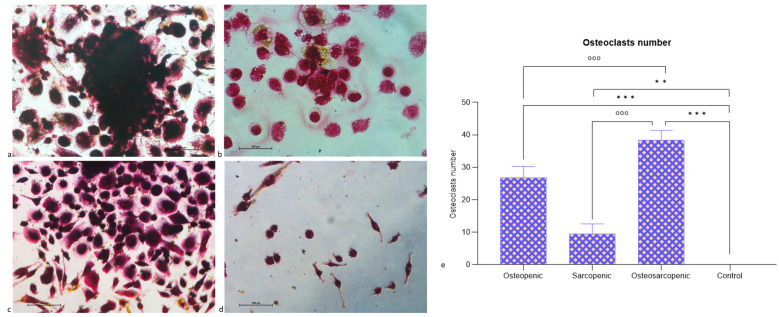
Multinucleated TRAP-positive osteoclasts after 3 weeks of culture. (**a**) OP, (**b**) SP, (**c**) OS, and (**d**) Control patients. TRAP-positive (dark purple) cells with at least three nuclei were considered osteoclasts. Magnification 20×. (**e**) Number of multinucleated TRAP-positive osteoclasts (three or more nuclei) evaluated in 10 regions of interest (ROI) after 3 weeks of cell culture at 20× magnification (Mean ± SD). One-way ANOVA followed by Dunnett’s test of OP, OS, and SP vs. Control (*) and OP and SP vs. OS (°): 2 symbols, *p* < 0.005, 3 symbols, *p* < 0.0005. Scale bar 100 µm.

After three weeks, TRAP staining revealed the presence of multinucleated TRAP-positive osteoclasts for OP, SP, and OS patients (Figure 4a–c) in comparison to Control patients (Figure 4d). OS patients showed a significantly higher number of osteoclasts compared to OP (*p* < 0.0005), SP (*p* < 0.0005), and Control (*p* < 0.0005) patients (Figure 4e). Furthermore, OP (*p* < 0.0005) and SP (*p* = 0.001) patients also showed a significantly higher number of osteoclasts compared to Control patients (Figure 4e).

### 2.4. Histology and Histomorphometry

**Figure 5:**
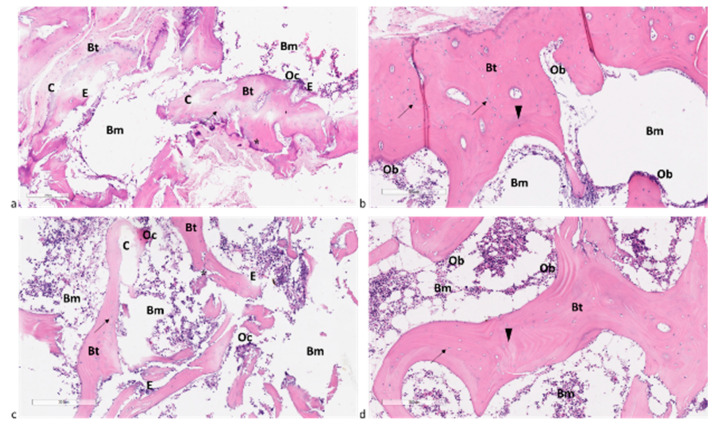
Histological findings in bone biopsy from (**a**) OP, (**b**) SP, (**c**) OS, and (**d**) Control patients. Hematoxylin/Eosin staining. Magnification 8×. Osteoporotic cavities (C), bone marrow spaces (Bm), bone trabeculae (Bt), osteocytes (↑), cement lines (arrowhead), refractile areas (*), eroded areas (E), osteoblasts (Ob), and osteoclast (Oc). Scale bar 300 µm.

Histological sections of bone biopsies from the Control and SP patients demonstrated a normal histological appearance, showing regular trabecular bone with bone marrow spaces in between (Figure 5b,d). Additionally, the classical appearance of bone with numerous Haversian systems covered by periosteum was observed (Figure 5b,d). Osteoprogenitor cells, osteoblasts, osteocytes, and cement lines were also visible (Figure 5b,d). Sections from the OP and OS patients revealed a loss of normal architecture in the trabecular bone, with thin, disconnected, and widely separated trabeculae (Figure 5a,c). Some trabeculae exhibited refractile areas indicating bone loss and necrosis (Figure 5a,b). Eroded areas were also observed on the bone surface (Figure 5a,b). Furthermore, some osteocytes had wide lacunae, with an apparent decrease in their number compared to the Control and SP sections (Figure 5a,b). Several resorption cavities were evident within the matrix (Figure 5a,b). Areas of the palely stained osteoid matrix, as well as multinucleated osteoclasts housed within erosion cavities, were also noted (Figure 5a,b).

**Figure 6:**
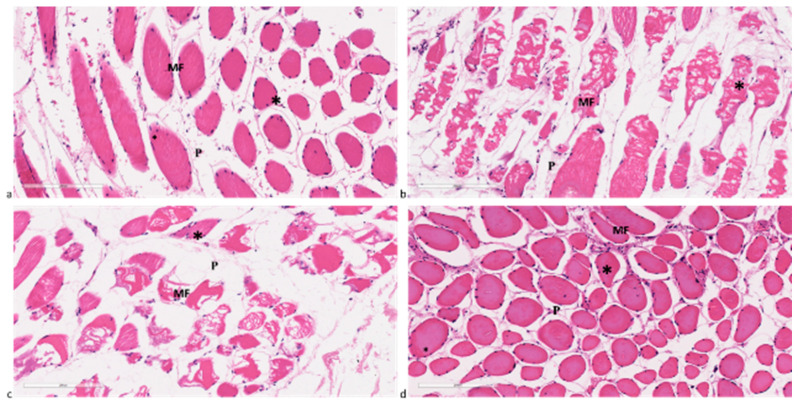
Histological findings in muscle biopsies from (**a**) OP, (**b**) SP, (**c**) OS, and (**d**) Control patients. Nuclei (star), myofibers (MF), perimysium (P). Hematoxylin/Eosin staining. Magnification 20×. Scale bar 200 µm.

Concerning muscle fibers, OP and Control patients did not exhibit any histological alterations (Figure 6a,d). In these cases, individual muscle fibers were bound together in bundles or fascicles. Within each bundle, each myofiber was connected to the others by a fine web of collagenous connective tissue (perimysium), which surrounds each myofiber. The skeletal muscle fibers are multinucleated with nuclei that are elongated or oval and located immediately beneath the sarcolemma. In contrast, muscle fibers from SP and OS patients displayed histological damage, such as focal degeneration and fibrosis (Figure 6b,c). Additionally, an increase in collagenous connective tissue infiltration in the perimysium, along with centrally located nuclei, was also observed.

In terms of bone histomorphometric results, Table 4 reports measurements for bone volume/tissue volume (BV/TV) (%), trabecular thickness (Tb.Th) (µm), trabecular number (Tb.N) (1/mm), and trabecular separation (Tb.Sp) (µm) and confirms the histological appearance.

BV/TV was significantly lower in OP, SP, and OS patients compared to the Control group (*p* < 0.0005) and higher in SP patients than OS ones (*p* < 0.0005). Concerning Tb.Th, a similar trend was observed with lower values in OP, SP, and OS groups compared to the Control group. Furthermore, SP patients exhibited significantly higher Tb.Th compared to OS patients (*p* < 0.005). Tb.N reveals a significantly lower value in the OP group than the OS group (*p* < 0.05). In contrast, Tb.Sp showed significantly higher values in OP (*p* < 0.0005) and OS (*p* < 0.005) patients compared to the Control group (*p* < 0.0005). OP patients also showed significantly higher values compared to OS patients (*p* < 0.005).

**Figure 7:**
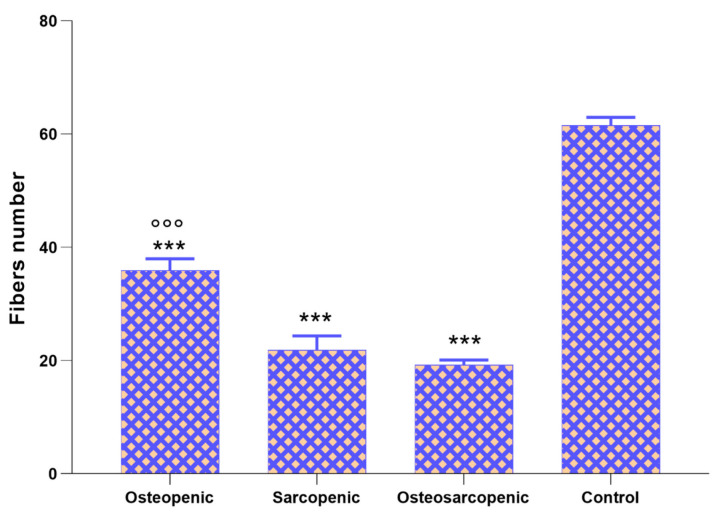
Number of muscle fibers in muscle biopsies of OP, SP, OS, and Control patients (Mean ± SD). One-way ANOVA (F = 278, *p* < 0.0005) followed by Dunnett’s test of OP, SP, and OS, vs. Control (*) and OP and SP vs. OS (°): 3 symbols, *p* < 0.0005.

Finally, the number of muscle fibers (Figure 7) was significantly lower in OP, SP, and OS patients compared to Control patients (*p* < 0.0005) and in OS patients compared to OP patients (*p* < 0.0005).

### 2.5. Immunohistochemistry

**Figure 8:**
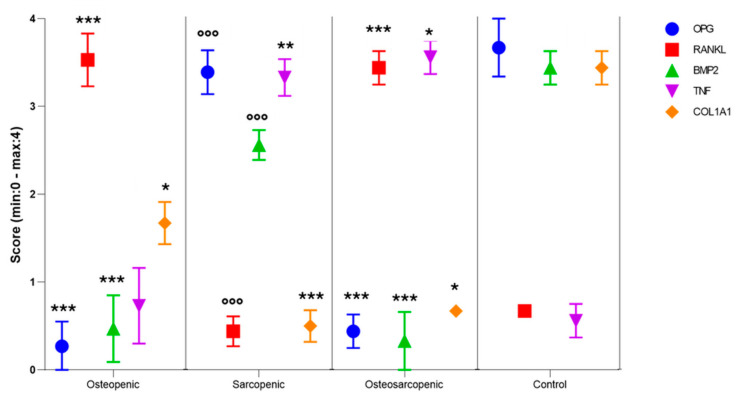
Dot plot of the results of De la Torre’s score for OPG, RANKL, BMP2, TNF, and COL1A1 immunostaining of OP, SP, OS, and Control patients (Median ± SE). One-way ANOVA followed by Dunnett’s test of OP, SP, and OS, vs. Control (*) and OP and SP vs. OS (°): 1 symbol, *p* < 0.05, 2 symbols, *p* < 0.005, 3 symbols, *p* < 0.0005.

Sections of bone biopsies were immunostained for OPG, RANKL, and BMP-2 (Supplementary File—Figure S1) while sections of muscle biopsies were immunostained for TNFα and COL1A1 (Supplementary File—Figures S2 and S3). The semi-quantification of their expression was carried out using De la Torre’s score (Figure 8). The score indicated significantly lower OPG (*p* < 0.0005) and BMP2 (*p* < 0.0005) positivity in the OP and OS patients compared to the Control group. In addition, SP patients exhibited significantly higher OPG and BMP2 levels compared to OS patients (*p* < 0.0005). In contrast to OPG and BMP2 levels, RANKL showed significantly higher values in OP and OS patients compared to the Control group (*p* < 0.0005), and lower values in SP patients compared to OS patients (*p* < 0.0005). Concerning muscle biopsies, the de la Torre score revealed significantly higher TNF positivity in SP and OS groups compared to the Control group (*p* < 0.0005). Finally, COL1A1 showed significantly lower values in OP (*p* < 0.05), SP (*p* < 0.0005), and OS (*p* < 0.05) patients compared to Control patients.

### 2.6. Linear Discriminant Analysis (LDA)

Most of the analyses and related results of LDA are reported in the Supplementary File (Figure S4 and Table S2). Among the blood parameters showing significant differences for each of the two types of comparisons, those with at least three significant correlations with histomorphometric and/or immunohistochemical parameters were selected as predictors for LDA: IL15, FGF2, TNFα, alpha-KLOTHO, DEHA-S, and spontaneous osteoclastogenesis count (#OC). According to the identified multicollinearities, LDA was conducted on scaled predictors in the following models: (1) IL15 + TNFα; (2) DEHA-S + TNFα; (3) #OC + IL15; (4) #OC + Alpha-KLOTHO; and (5) #OC + DEHA-S. The accuracy in classification was 78% for model 1, 72% for model 2, 67% for models 3 and 4, and 61% for model 5. The highest percentages of correctly classified (≥80%) were achieved for the Control group with models 1–3, OP patients with models 1 and 5, SP patients with model 2, and OS patients with models 3 and 4.

Except for #OC, the other predictors did not show a univariate normal distribution and it was necessary to perform a logarithmic (IL-15, FGF2, and Alpha-KLOTHO), quadratic (TNFα), or Box-Cox (DEHA-S) transformation. None of these predictors had outliers. Having then identified multicollinearity between FGF2 and all the other predictors, between IL-15 and Alpha-KLOTHO and DEHA-S, and between #OC and TNFα, LDA was conducted on the scalar predictors in the following models by associating them with the ranking factor (Groups): (1) IL-15 + TNFα; (2) DEHA-S + TNFα; (3) #OC + IL-15; (4) #OC + Alpha-KLOTHO; and (5) #OC + DEHA-S. For each model training, Table S4 reports the linear discriminant coefficients and the proportion of trace that represents the proportion of between-class variance that is explained by successive discriminant functions.

Since there were not enough patients to be able to split the dataset into 70% cases for training and 30% for testing in order to conduct cross-validation of the models, after conducting the training phase of the models on the entire dataset, the testing phase was conducted using a new dataset with the same number of patients randomly generated by considering the correlation values r between the predictors of the original dataset. For each model, Figure S4 summarizes the results of the testing phase of the cross-validation process by reporting the classification accuracy values and specifying the percentage of correct classifications for each model and group.

## 3. Discussion

In the present study, we explored circulating, cellular, and tissue biomarkers in patients with osteopenia, sarcopenia, and osteosarcopenia and Control subjects affected by degenerative conditions of the lumbosacral spine undergoing posterior vertebral fusion surgery. The aim was to identify potential innovative and distinctive biomarkers representative of osteosarcopenia. Our results revealed novel findings specific to osteosarcopenic patients. Firstly, an elevated basophil percentage and TNFα were observed, along with decreased aPPT, INR, IL15, alpha-Klotho, DEHA-S, and FGF2 expression compared to all other patient groups. Secondly, for the first time, significantly higher spontaneous osteoclastogenesis was detected in osteosarcopenic patients compared to osteopenic, sarcopenic, and Control patients. Thirdly, like osteopenic and sarcopenic patients, those with osteosarcopenia exhibited a lower neutrophil percentage, increased IL6, IL1β, and PGE2 expression, and reduced IL33, IL10, ST2, and CTNF levels compared to the Control group. Lastly, osteosarcopenic patients displayed distinctive bone and muscle tissue structure, morphology, and microarchitecture, as well as characteristic immunoreactivity for OPG, RANKL, BMP-2, TNFα, and COL1A1, as detected, for the first time, by histological, immunohistochemical, and histomorphometrical analyses on bone and muscle biopsies.

In various studies, a consistent link has been found between sarcopenia and elevated levels of pro-inflammatory mediators, particularly soluble TNFα receptors, in comparison to older individuals without sarcopenia [15,16]. The administration of TNFα has been shown to induce muscle atrophy and loss through several pathways such as nuclear factor kappa-light-chain-enhancer of activated B (NF-κB) activation and mammalian target of rapamycin (mTOR) suppression [17,18]. Similar increases in TNFα levels have been observed in the context of osteopenia/osteoporosis, where TNFα plays various roles, including inducing RANKL expression, activating osteoclast transcription factors, inhibiting pre-osteoblast maturation and osteoblast activity, inducing osteoblast apoptosis, and inhibiting genes involved in bone formation [19]. Therefore, our results demonstrate that the upregulation of TNFα, leading to both aging of muscle cells and deterioration of skeletal muscle, has an additive effect in osteosarcopenic patients. In addition to TNFα, there are other muscle-derived endocrine factors and hormones such as FGF2, IL6, IL15, and DHEA-S that can exert some biological activity in bone cells [20]. Here, we detected decreased levels of IL15, FGF2, and DEHA-S in osteosarcopenic patients compared to all the other groups. In contrast to TNFα, which contributes to muscle atrophy, IL15, a myokine released by skeletal muscle, is identified as an anabolic factor in muscle growth. Several studies have reported that reduced IL15 levels are a common risk factor for sarcopenia, obesity, and immunosenescence [21]. In fact, IL15 plays a role in reducing adipose tissue mass and the progression and survival of NK lymphocytes. Furthermore, IL15 has an anti-apoptotic ability through the inhibition of pathways mediated by TNFα [21,22,23]. In contrast to the role of IL15 in sarcopenia, some evidence suggests that IL15 could promote the activation of osteoclasts, thus contributing to bone resorption and BMD loss during osteoporosis [24,25]. However, in our study, similar IL15 levels were found among osteoporotic and sarcopenic patients, while significantly lower levels were detected exclusively in osteosarcopenic patients. These data highlight how in the presence of both pathological conditions, this interleukin plays an individual role, suggesting an exclusive interaction between the two tissues. As previously highlighted, in muscle–bone crosstalk, skeletal muscle can secrete hundreds of myokines to regulate bone metabolism positively or negatively, and in this context, the FGF family is of critical importance [26]. FGFs have many cellular functions related to processes such as proliferation, differentiation, and survival [26,27]. FGF2 plays a critical role in adult regenerative myogenesis; however, the exact role of FGF2 in sarcopenia is undefined [26]. It was reported that FGF2 also facilitates bone formation mediated by the modulation of the Wnt/β-catenin signaling pathway [26]. In addition, Xiao et al. showed that the overexpression of FGF2 in osteoblasts and osteocytes enhances the repair of critical-size bone defects in mice [27]. All these data suggest that muscle represents a determining factor of bone loss and that paracrine signaling via local growth factors including FGF2 between muscle and bone could be of key importance. In addition to FGF2, other factors such as hormones such as DHEA-S, the adrenal-derived hormone dehydroepiandrosterone sulfate ester, could also play a key role in muscle and bone crosstalk. Literature data suggest that elevated DHEA-S levels are associated with a reduced risk of sarcopenia and protection against BMD loss [28,29]. Postulated mechanisms for muscle and BMD control by DHEA-S include its conversion to androgen and estrogen, alterations in hormone-receptor sites, and the regulation of enzymatic activity, all of which are plausibly related to muscle and bone metabolism [30,31]. It is reasonable to presume from our study that low DHEA-S levels increase the likelihood of osteosarcopenia, despite the lack of previous studies showing a relationship between the two.

Among endocrine factors, α-Klotho, originally identified as a senescence-related protein and involved in the regulation of various metabolic processes in humans [32], was evaluated for the first time in osteosarcopenic patients, showing a significant decrease in these patients compared to all the other groups. In the existing literature, it is already known that α-Klotho deficiency leads to an aging-like phenotype with sarcopenia, metabolic disorders, osteoporosis, impaired cognition, gait disturbance, and atherosclerosis [33,34]. However, to the best of our knowledge, this is the first study that associates this protein with osteosarcopenia. Due to its various roles in aging, metabolism, and disease, α-Klotho is considered today a potential therapeutic target, and several researchers are exploring ways to modulate its expression or activity to potentially mitigate age-related diseases [35,36]. Therefore, the modulation of Klotho could open the door to novel interventions aimed at addressing the challenges also for osteosarcopenia.

All this evidence suggested not only the exclusive interactions occurring between the two tissues but also the shared physiopathology of osteoporosis and sarcopenia through common molecular factors, hormonal imbalance, and increased cytokine activity.

Another innovative and novel aspect of our study was the evaluation of spontaneous osteoclastogenesis in osteosarcopenic patients. The results demonstrated a significantly higher occurrence of spontaneous osteoclastogenesis in osteosarcopenic patients compared to all the other groups. Previous studies have established that the spontaneous differentiation of PBMCs into osteoclasts in vitro, without the addition of exogenous factors, serves as a predictive factor for patients affected by local or systemic bone remodeling diseases, such as osteoporosis/osteopenia. However, no studies have demonstrated spontaneous osteoclastogenesis in osteosarcopenic patients [37,38,39]. This phenomenon was associated with a link between the immunoregulation by T cells and spontaneous osteoclast formation, with increased levels of TNFα and RANKL [40]. Specifically, TNFα stimulates RANKL expression in osteoblasts and tissue stromal cells, promoting osteoclast differentiation and activity [41]. These findings align with the results we obtained, not only for spontaneous osteoclast formation but also for TNFα, which was found to be more upregulated in osteosarcopenic patients. In vivo studies have demonstrated that in the presence of acute muscle paralysis, there is a rapid upregulation of RANKL in the bone microenvironment, which has been associated with an increase in the number of osteoclasts [42,43,44,45]. These findings provide an additional perspective to consider how muscles and bones cooperate to maintain skeletal homeostasis and highlight the essential coupling between muscle health and bone quality.

To explore the relationship between blood cell composition and osteosarcopenia and to speculate a feasible index for the auxiliary diagnosis, changes in hematological parameters were also analyzed. In this study, percentages of basophils, INR, and aPPT were significantly different among osteoporotic, sarcopenic, Control, and osteosarcopenic patients, even though they were all within the normal range. Because these factors fall within the normal range, it is not possible to predict their connection with the condition of osteosarcopenia, even considering that all these patients have comorbidities and are undergoing multi-drug therapy.

In addition to the previously discussed findings, in individuals with osteosarcopenia, like those with osteopenia and sarcopenia, there were notable differences compared to the Control group. These included a lower neutrophil percentage, increased expression of IL6, IL1β, and PGE2, and reduced levels of IL33 and its receptor ST2, IL10, and CTNF. The lower neutrophil percentage in osteopenic, sarcopenic, and osteosarcopenic patients may be a reactive response influenced by various factors, including age. Pro-inflammatory cytokines, such as IL6 and IL1β, contribute significantly to the phenomenon of inflammation, both during sarcopenia and osteoporosis, also enhancing PGE2 [46,47]. PGE2, associated with osteoclastogenesis, contributes to bone resorption in osteoporosis [48]. CNTF, belonging to the IL6 family, plays a trophic role in muscle tissues and in bone metabolism [49,50]. Additionally, reduced levels of IL10 and IL33, cytokines linked to immune cells, were observed in patients with these conditions, impacting osteoclast formation and bone matrix mineralization [51].

Furthermore, osteosarcopenic patients exhibited distinctive morphology and microarchitecture in bone and muscle tissues, along with characteristic immunoreactivity for OPG, RANKL, BMP-2, TGFα, and COL1A1. Bone biopsies from osteosarcopenic and osteopenic patients displayed severe trabecular bone loss, whereas sarcopenic and Control patients showed a relatively intact trabecular structure, as demonstrated by the bone volume value and trabecular separation and thickness results. Muscle biopsies from osteosarcopenic and sarcopenic individuals revealed reduced muscle tissue quality and number, marked by the replacement of muscle fibers with fat and an increase in fibrosis. Immunohistochemistry further confirmed these results, showing a significantly higher positivity for RANKL (in bone) and TGFβ (in muscle), along with reduced positivity for BMP2, OPG (in bone), and COL1A1 (in muscle) in osteosarcopenic patient biopsies, confirming these distinct characteristics for the first time.

Finally, we reported an LDA model based on the changes in six biomarkers in different groups. A classification accuracy ≥ 80% was achieved for osteosarcopenic patients with the model considering the quantification of spontaneous osteoclastogenesis associated with IL15 expression or alpha-Klotho expression. These results may have potential clinical implications for guiding the choice of specific biomarkers for osteosarcopenia.

This study has identified several innovative biomarkers for osteosarcopenia. However, it has some limitations. The sample size was inadequate, despite being the first to investigate circulating, cellular, and tissue biomarkers for osteosarcopenia. Our findings require validation in a future prospective study with a larger sample size to achieve a more accurate classification model. Additionally, important bone and muscle biomarkers and growth hormones were not analyzed. Finally, it is important to note that our study involved patients with degenerative conditions of the lumbosacral spine who underwent posterior vertebral fusion surgery. Therefore, it is necessary to exercise caution when generalizing the findings of this study.

## 4. Materials and Methods

### 4.1. Patients Population

Eighteen consecutive patients affected by degenerative conditions (adult scoliosis, stenosis, disc herniation, and spondylolisthesis) of the lumbosacral spine undergoing posterior vertebral fusion surgery from the age of 40 were recruited. Fresh bone and muscle samples and blood samples were obtained from each patient undergoing vertebral fusion surgery. Exclusion criteria were patients with spine conditions of tumor or post-traumatic origin; patients with a history of musculoskeletal infections; and patients who had previously undergone spinal surgery.

The pilot sample size was calculated as at least 25% of the total sample size achieved by a priori power analysis using G*Power software. (v.3.1.9.7) [52]. The calculation was carried out by selecting F-tests MANOVA and considering an effect size f^2^ = 0.16, a power of 0.80, and an α-value of 0.05 for 4 groups (OP, SP, OS, and Control) and at least 14 response variables.

### 4.2. Baseline Characteristics, Demographics, and OS Definition

Demographic and clinical data, such as age, gender, BMI, length of stay, therapy/drugs used, magnetic resonance image (MRI) findings, and blood tests before surgery (white blood cells, red blood cells, hemoglobin, hematocrit, MCV, MCH, MCHC, RDW (%), RDW DV, neutrophils, lymphocytes, monocytes, eosinophils, basophils, platelets count, MPV, prothrombic activity ratio, prothrombic activity INR, aPPT, and C-reactive protein), as well as comorbidities assessed using the Elixhauser Comorbidity Index (ECI) and polypharmacy, were collected. Central sarcopenia and osteopenia were evaluated for every patient through MRI, measuring PLVI [53] and the M-score [54], respectively:

- PLVI was determined by dividing the average cross-sectional area (CSA) of the psoas muscle by the average area of the L4 vertebrae, PLVI = (left psoas CSA + right psoas CSA)/(2 × L4 vertebral body CSA), as described previously [55,56].
- The M-score was calculated based on the T1W spin-echo sequence of the preoperative MRI. The M-score was derived using the mean and standard deviation of a reference population, as follows: M-Score = (Signal-to-Noise Ratio (SNR) L1-L4—SNRref)/SDref [54].

Patients were stratified according to the mean values of PLVI and the M-score (PLVI = 0.85; M-score = 2.67). Those with PLVI and M-score values below the average were categorized as SP and/or OP.

### 4.3. Serum Measurement

For serum preparation, peripheral venous blood samples (~2–3 mL) were centrifugated at 2500 rpm for 15 min. The serum was aliquoted and stored at −80 °C until further testing. Serum IL-15 (lot. L230418301, Cloud-Clone, Katy, TX, USA), IL-6 (lot. 341574-010, Invitrogen, Waltham, MA, USA), TNF-α (lot. 20230327, Bio-Techne, Minneapolis, MN, USA), IL-1β (lot. 20230328, Bio-Techne, MN, USA), IL-10 (lot. 40001589, Bio-Techne, MN, USA), IL-33 (lot. 20230412, Bio-Techne, MN, USA), α-Klotho (lot. HAK0423, Bio-Techne, MN, USA), ST2 (lot. EB24344, Bio-Techne, MN, USA), CNTF (H04971043Y, Bio-Techne, MN, USA), DHEA-S (lot. 3E265N, Bio-Techne, MN, USA), FGF2 (lot. L230505211, Cloud-Clone, TX, USA), and PGE2 (lot. L230505211, Cloud-Clone, TX, USA) were measured in triplicate using ELISA kits.

### 4.4. Spontaneous Osteoclastogenesis

To evaluate spontaneous osteoclastogenesis, the PBMCs were obtained from peripheral blood samples (~2 mL) according to the Ficoll (Sigma-Aldrich, St. Louis, MO, USA) method. Briefly, a volume of peripheral blood was diluted 1:1 with phosphate-buffered saline (PBS, Sigma-Aldrich, MO, USA) and layered on Histopaque 1077 (ratio 2:1). Density gradient centrifugation (700 g for 30 min) was used to separate the PBMCs from the other cell fractions of blood. After centrifugation, the PBMCs at the interface between PBS and Ficoll were collected, washed, resuspended in Dulbecco’s modified Eagle’s medium (DMEM; Sigma-Aldrich, MI, USA) + 10% fetal calf serum (FCS; Lonza, Verviers, Belgium), counted, and seeded at a density of 1.5 × 10^6^/cm^2^. Three culture days later, the non-adherent cells were removed, and adherent PBMCs were examined using a light microscope (Olympus IX 71) and images were acquired using a digital image capture system (×40 objective and an Olympus XC camera) (Boston Industries, Inc. 10 Industrial Rd. Walpole, MA 02081, USA). After 1, 2, and 3 weeks of PBMC culture, the Alamar blue dye test (Serotec, Oxford, UK) was used to evaluate cell viability. The reagent is a dye, which incorporates an oxidation-reduction indicator that changes color in response to a chemical reduction in the growth medium resulting from cell growth. It was added to each culture well (1:10 *v*/*v*) for 4 h at 37 °C. After transferring the supernatants to 96-well plates, the absorbance was read spectrophotometrically at 570- and 600-nm wavelengths by a MicroPlate reader (BioRad, Hercules, CA, USA). The results, obtained as optical density (OD), were processed following the manufacturer’s instructions and expressed as reduction percentages.

After 3 weeks of cell culture, the differentiation of PBMC in osteoclasts was evaluated using TRAP histochemical staining according to the manufacturer’s instructions (Sigma, St. Louis, MO, USA). The large, multinucleated cells (three or more nuclei) that developed a dark purple color were scored as positive cells. Images were taken using a standard light microscope (Olympus IX71) equipped with a digital camera (XCell, Olympus Italia Srl, Segrate Milano, Milano, Italy) at ×20 magnification. In addition, the number of osteoclasts, in 10 ROI at ×20, was also calculated.

### 4.5. Histology and Histomorphometry

Bone and muscle samples were placed in 10% formalin buffer and processed for paraffin embedding procedures. Bone samples were decalcified in a 5% solution of formic and hydrochloric acid for approximately 48 h. Subsequently, bone and muscle samples were extensively rinsed in distilled water, dehydrated in graded alcohol solutions, cleared in xylene, and finally, paraffin-embedded. Sections (5 ± 1 μm) were taken by a semi-automated microtome (HM Leica microtome, Bologna, Italy), and three slides for each sample were stained with hematoxylin and eosin (H&E). Histological images were taken with a digital pathology slide scanner (Aperio-Scanscope, Leica Biosystems, Nußloch, Germany).

Histomorphometric measurements were performed using Aperio eSlide Manager software v12.4.3.5008 (Aperio-Scanscope, Leica Biosystems, Nußloch, Germany). The analyses were carried out on the whole image at 10× magnification. Histomorphometric parameters for bone samples were calculated as defined by the “American Society of Bone and Mineral Research (ASBMR)” [57]. In detail, for each bone section, BV/TV (%), Tb.N (1/mm), Tb.Sp (mm), and Tb.Th (mm) were assessed.

For muscle samples, the proportion of fibers (relative distribution of fibers in the field) was assessed by counting fibers in 5 random fields in both muscle histologies.

### 4.6. Immunohistochemistry

Two sections of bone and muscle biopsies for each sample were dewaxed in decreasing-grade ethanol solutions until PBS rinsing. Subsequently, the two sections of bone biopsies were immunostained for OPG (lot. sc-390518, Santa Cruz Biotechnology, Inc., Dallas, TX, USA), RANKL (lot. bs-0747R, Bioss Antibodies, Woburn, MA, USA), and BMP2 (lot. MSA7121012, R&D System, Minneapolis, USA). Meanwhile, the two sections of muscle biopsies were immunostained for TNFα (lot. A20220916558, Cloud-Clone, TX, USA) and COL1A1 (lot. sc-293182, Santa Cruz Biotechnology, Inc., Dallas, TX, USA). Briefly, after fixation, sections were rinsed in PBS, permeabilized with 0.3% hydrogen peroxide, and pre-treated for antigen unmasking with 0.2% Pronase (Sigma-Aldrich, Saint Louis, MO, USA). Then, 10% normal serum was added to block nonspecific antibody binding and the primary antibodies were applied and incubated overnight. After rinsing in PBS, slides were incubated with appropriate biotinylated secondary antibodies and horseradish peroxidase-streptavidin complex (Bethyl Laboratories, Inc., Montgomery, TX, USA). The sample reaction was developed with a 3,3-diaminobenzidine substrate and permanently mounted. Negative controls, by omitting the primary antibody, were included to check the proper specificity and performance of the applied reagents.

Staining assessment for immunostained sections was performed in bone and muscle biopsies as reported by de la Torre et al. [58] using Aperio eSlide Manager software (Leica Biosystems, Bologna, Italy). Briefly, three regions of interest were scored at 40× magnification; the percentage of positive cells was evaluated with a manual count. For each region of interest, a value of 0 to 4 (0: negative; 1: <5% of cells with positive staining; 2: between 5 and 50% of cells with positive staining; 3: more than 50% of cells with weak staining; 4: more than 50% of cells with strong staining) was assigned for OPG, RANKL, BMP2, TNFα, and COL1A1.

### 4.7. Linear Discriminant Analysis (LDA)

The objective of this preliminary LDA was to identify, among the blood parameters that had shown significant differences for both types of comparisons (OP, SP, and OS groups vs. Control group, and among OP and SP vs. OS), those that are able to correctly classify the sample of patients into the 4 categories of OP, SP, OS, and Control. The blood parameters that were considered in the subsequent analyses were %Basophils, INR, aPTT, IL-15, TNF-α, FGF2, Alpha-KLOTHO, DEHA-S, and spontaneous osteoclastogenesis in termid OC counts (#OC).

Firstly, a correlation analysis was conducted among these blood parameters and the histomorphometric (BV/TV, Tb.Th, Tb.N, Tb.SP, and #Fibers) and immunohistochemical (OPG, RANKL, BMP2, TNF-a, and COL1A1) parameters to select the blood parameters that had at least three or more significant correlations (|r| ≥ 0.65, *p* < 0.005).

### 4.8. Statistical Analysis

Statistical analysis was performed using R software (v.4.3.2) [59]. Demographic and clinical data are reported as Means and 95% Confidence Intervals (CIs) while the results of assays are reported as Means ± standard deviations (SD) or Medians ± standard error (SE) at a significance level of *p* < 0.05. After having verified the normal distribution and homogeneity of variance, a one-way or two-way ANOVA (viability test) was performed to compare data among patient groups (OP, SP, OS, and Control). Finally, the post hoc Dunnett’s test was performed to detect significant differences among OP, SP, and OS groups vs. the Control group, and among OP and SP vs. OS.

Subsequently, the blood parameters that showed significant differences in both types of comparison described above were selected to check their correlation with the histomorphometric and immunohistochemical parameters, and then those presenting equal or more than three correlations were used in the linear discriminant analysis (LDA) to find their linear combination that gave the best possible separation among the groups. LDA was conducted using the R package MASS (v.7.3-60) [60]; before proceeding to LDA, the most critical assumptions for conducting it (e.g., normality, no outliers, and multicollinearity) were verified.

## 5. Conclusions

To conclude, we explored biomarkers of osteosarcopenia in patients affected by degenerative conditions of the lumbosacral spine undergoing posterior vertebral fusion surgery. The results showed that elevated TNFα levels and spontaneous osteoclastogenesis increased the likelihood of osteosarcopenia and decreased IL15, alpha-Klotho, DEHA-S, and FGF2 levels. Furthermore, osteosarcopenic patients displayed distinctive bone and muscle tissue structure, morphology, and microarchitecture, as well as characteristic immunoreactivity for OPG, RANKL, BMP-2, TNFα, and COL1A1, as detected by histological, immunohistochemical and histomorphometric analyses on bone and muscle biopsies. These findings are novel as only a few of the parameters we identified have been previously reported as biomarkers of osteosarcopenia. These results are of key importance since accurately and consistently measuring various aspects of osteosarcopenia remains challenging due to the inherent limitations of current assessment tools, such as their lack of specificity and variability based on different population characteristics.

## Figures and Tables

**Table 1:** Baseline characteristic differences for osteopenic (OP), sarcopenic (SP), osteosarcopenic (OS), and healthy Control patients. Data are reported as the Mean [95% CI] or n (%).

Characteristics	Total (n = 18)	OP (n = 5)	SP (n = 6)	OS (n = 4)	Control (n = 3)
**Sex (female %)**	67%	11%	28%	22%	6%
**Age at surgery (years)**	63.9 [59.8, 69.1]	64.4 [56.3, 72.5]	68.0 [60.4, 75.6]	65.8 [61.2, 70.3]	52.7 [42.9, 62.4]
**Age Class**	**n (%)**	**n**			
40–60	6 (33.3)	2	1	1	2
>60	12 (66.7)	3	5	3	1
**BMI**	25.19 [23.8, 26.6]	28.65 [26.73, 30.57]	23.88 [21.57, 26.20]	23.38 [21.30, 25.45]	24.43 [20.21, 28.66]
**BMI Class**	**n (%)**	**n**			
Healthy weight 18.5–24.9	10 (55.6)	0	5	3	2
Overweight 25.0–29.9	6 (33.3)	3	1	1	1
Class 1 Obesity 30.0–34.9	2 (11.1)	2	0	0	0
**PLVI**	0.83 [0.69, 0.98]	1.23 [0.86, 1.60]	0.69 [0.64, 0.74]	0.50 [0.49 0.51]	0.89 [0.71, 1.09]
**M-score**	2.62 [2.14, 3.09]	1.54 [1.07, 2.01]	3.71 [3.22, 4.20]	1.65 [1.08, 2.21]	3.51 [2.85, 4.17]
**Inpatient diagnosis**	**n (%)**	**n**			
Lumbar disc herniation	4 (22.2)	2	1	0	1
Lumbar spondylolisthesis	2 (11.1)	0	0	1	1
Lumbar/lumbosacral stenosis	10 (55.7)	3	4	2	1
Wound dehiscence in lumbosacral arthrodesis	1 (5.5)	0	0	1	0
Thoraco-lumbosacral idiopathic kyphosis	1 (5.5)	0	1	0	0
**Length of stay (day)**	12.75 [9.35, 16.15]	16.50 [9.09, 23.91]	9.50 [7.59, 11.41]	19.33 [1.81, 36.85]	7.66 [1.95, 13.37]

**Table 2:** Hematological parameters for osteopenic (OP), sarcopenic (SP), osteosarcopenic (OS), and healthy Control patients. Data are reported as Means [95% CI]. One-way ANOVA followed by Dunnett test of OP, OS, and SP vs. Control (*) and OP and SP vs. OS (°): 1 symbol, *p* < 0.05, 2 symbols, *p* < 0.005.

Blood Parameters	Reference Values	Total (n = 18)	OP (n = 5)	SP (n = 6)	OS (n = 4)	Control (n = 3)	F, *p*
**Neutrophils**	42–77%	53.8 [46.5, 61.0]	56.9 * [48.1, 65.8]	43.8 ** [39.1, 48.5]	42.5 ** [28.3, 56.6]	83.6 [69.8, 97.3]	8.36, 0.002
**Basophils**	0–1.8%	0.44 [0.32, 0.56]	0.44 ° [0.27, 0.59]	0.30 °° [0.11, 0.49]	0.85 * [0.68, 1.02]	0.20 [0.21, 0.22]	6.30, 0.006
**INR**	<1.2	1.03 [1.01, 1.06]	1.04 ° [1.01, 1.07]	1.05 ° [1.02, 1.08]	0.95 ** [0.91, 0.99]	1.10 [1.04, 1.16]	6.34, 0.006
**aPPT**	0.82–1.25	0.95 [0.90, 1.01]	0.99 ° [0.90, 1.08]	1.00 ° [0.93, 1.07]	0.80 * [0.68, 0.91]	1.02 [0.95, 1.08]	3.61, 0.040

**Table 3:** ECI and polypharmacy results for osteopenic (OP), sarcopenic (SP), osteosarcopenic (OS), and healthy Control patients. Data are reported as Means [95% CI].

	Total (n = 18)	OP (n = 5)	SP (n = 6)	OS (n = 4)	Control (n = 3)
**ECI**					
Score	−1.3 [−3.6, 0.9]	−3.6 [−6.7, −0.5]	−1.5 [−6.5, 3.6]	3.0 [−2.1, 8.1]	−3.0 [−4.6, −1.4]
	**n**				
<0	12	3	5	1	3
0	3	2	0	1	0
1 to 4	0	0	0	0	0
≥5	3	0	1	2	0
**Polypharmacy**					
Drugs taken	4.6 [3.2, 6.0]	5.0 [2.3, 7.7]	4.1 [1.4, 6.9]	5.3 [2.2, 8.3]	4.0 [0.2, 7.8]
	**n**				
‘no polypharmacy’ 0–4 drugs	10	2	5	1	2
‘polypharmacy’ > 5 drugs	6	3	0	2	1
‘excessive polypharmacy’ >10 drugs	2	0	1	1	0

**Table 4:** Results of histomorphometric parameters in bone biopsies of OP, SP, OS, and Control patients (Mean ± SD). One-way ANOVA followed by Dunnett’s test of OP, SP, and OS, vs. Control (*) and OP and SP vs. OS (°): 1 symbol, *p* < 0.05, 2 symbols, *p* < 0.005, 3 symbols, *p* < 0.0005.

Parameters	OP (n = 5)	SP (n = 6)	OS (n = 4)	Control (n = 3)	F, *p*
**BV/TV (%)**	25.2 ± 3.7 ***	55.2 ± 6.7 ***,°°°	33.5 ± 8.6 ***	81.0 ± 7.6	52.75, <0.0005
**Tb.Th, µm**	86 ± 9 ***	147 ± 33 *,°°	83 ± 11 ***	202 ± 27	21.59, <0.0005
**Tb.N, 1/mm**	0.29 ± 0.06 °	0.39 ± 0.08	0.42 ± 0.04	0.40 ± 0.02	4.51, 0.021
**Tb.Sp, µm**	262 ± 55 ***,°°	119 ± 30	166 ± 38 **	46 ± 17	22.21, <0.0005

## Data Availability

The datasets generated through this work are available in a publicly accessible repository: https://figshare.com/s/dd6cf961acdbd1fdeee4 (accessed on 3 April 2024).

## Supplementary Materials

The following supporting information can be downloaded at: https://www.mdpi.com/article/10.3390/ijms25115879/s1.
