# Supplementary material for: A Pilot Study on Circulating, Cellular, and Tissue Biomarkers in Osteosarcopenic Patients
Source: Int J Mol Sci. 2024 May 28;25(11):5879. doi: 10.3390/ijms25115879 (PMC11172451; doi:10.3390/ijms25115879)
Supplement: Supplementary file 1 [file ijms-25-05879-s001.zip › ijms-3014668-supplementary.pdf]

Supplementary File.

A PILOT STUDY ON CIRCULATING, CELLULAR AND TISSUE BIOMARKERS IN OSTEOSARCOPENIC PATIENTS. *Salamanna F et al.*

2. RESULTS

2.1 Demographics data and baseline clinical characteristics

Supplementary Table S1. Blood parameters for osteopenic (OP), sarcopenic (SP), osteosarcopenic (OS) and healthy control patients. Data are reported as mean [95% CI].

Blood parameters	Reference values	Total (n=18)	Osteopenic (n=5)	Sarcopenic (n=6)	Osteosarcopenic (n=4)	Control (n=3)
White blood cells	3.6-10.5 10^9/L	9.58 [8.73, 10.42]	7.77 [6.45, 9.08]	9.23 [8.41, 10.04]	10.66 [8.74, 12.58]	10.89 [8.41,13.38]
Red blood cells	3.85-5.20 10^{12}/L	3.89 [3.73, 4.05]	4.04 [3.67, 4.41]	3.79 [3.39, 4.18]	3.78 [3.50, 4.07]	4.00 [3.66, 4.34]
Hemoglobin	11.88-15.8 g/dL	11.54 [11.06, 12.03]	12.28 [11.09, 13.47]	11.8 [10.62, 12.98]	10.72 [10.10, 11.33]	11.63 [10.34, 12.92]
Hematocrit	35-45.5 %	33.72 [32.38, 35.06]	35.7 [32.53, 38.87]	34.33 [30.78, 37.88]	31.6 [30.07, 33.13]	33.83 [30.21, 37.45]
MCV	80-101 fL	86.99 [85.76, 88.23]	88.68 [87.11, 90.25]	90.67 [89.26, 92.07]	84.5 [81.61, 87.39]	84.27 [82.51, 86.02]
MCH	27-34 pg	29.76 [29.27, 30.25]	30.38 [29.85, 30.91]	31.23 [30.98, 31.49]	28.68 [27.47, 29.90]	28.93 [28.13, 29.74]
MCHC	31.5-36 g/dL	34.21 [33.99, 34.43]	34.36 [33.98, 34.74]	34.47 [34.17, 34.77]	33.85 [33.32, 34.38]	34.33 [33.82, 34.84]
RDW	11.5-15 %	14.18 [13.63, 14.72]	13.94 [13.53, 14.35]	13.37 [13.14, 13.59]	13.75 [13.20, 14.30]	16.5 [13.40, 19.60]
RDW DV	39-51 fL	44.68 [43.23, 46.14]	44.35 [42.69, 46.01]	44.33 [44.07, 44.60]	42.20 [40.84, 43.56]	53.5 [43.66, 63.34]
Lymphocytes	20-44 %	20.49 [17.92, 23.06]	29.21 [25.37, 33.04]	20.1 [17.7, 22.50]	18.14 [13.13, 23.15]	11.17 [4.59, 17.74]
Monocytes	2-9.5 %	5.99 [5.36, 6.63]	6.75 [5.03, 8.47]	7.25 [6.55, 7.95]	5.18 [4.23, 6.13]	4.67 [3.17, 6.16]
Eosinophils	0.5-55 %	2.33 [1.80, 2.85]	3.31 [1.95, 4.66]	1.85 [1.31, 2.39]	2.8 [1.87, 3.73]	0.40 [0.06, 0.74]
Platelets count	160-370 10^9/L	231 [216, 246]	219 [181, 257]	297 [269, 325]	195 [180, 212]	233 [220, 246]
MPV	8.5-11.5 fL	9.29 [8.67, 9.9]	7.34 [5.53, 9.15]	9.93 [9.74, 10.13]	10.25 [9.58, 10.92]	9.73 [8.29, 11.18]
Prothrombic activity ratio	<1.2	0.98 [0.94, 1.03]	0.87 [0.73, 1.01]	0.97 [0.96, 0.98]	1.03 [1.02, 1.050]	1.1 [1.06, 1.13]
C-reactive protein	<0.5 mg/dL	0.5 [0.24, 0.77]	1.62 [0.61, 2.64]	0.09 [0.08, 0.10]	0.15 [0.11, 0.18]	-

2.5 Immunohistochemistry

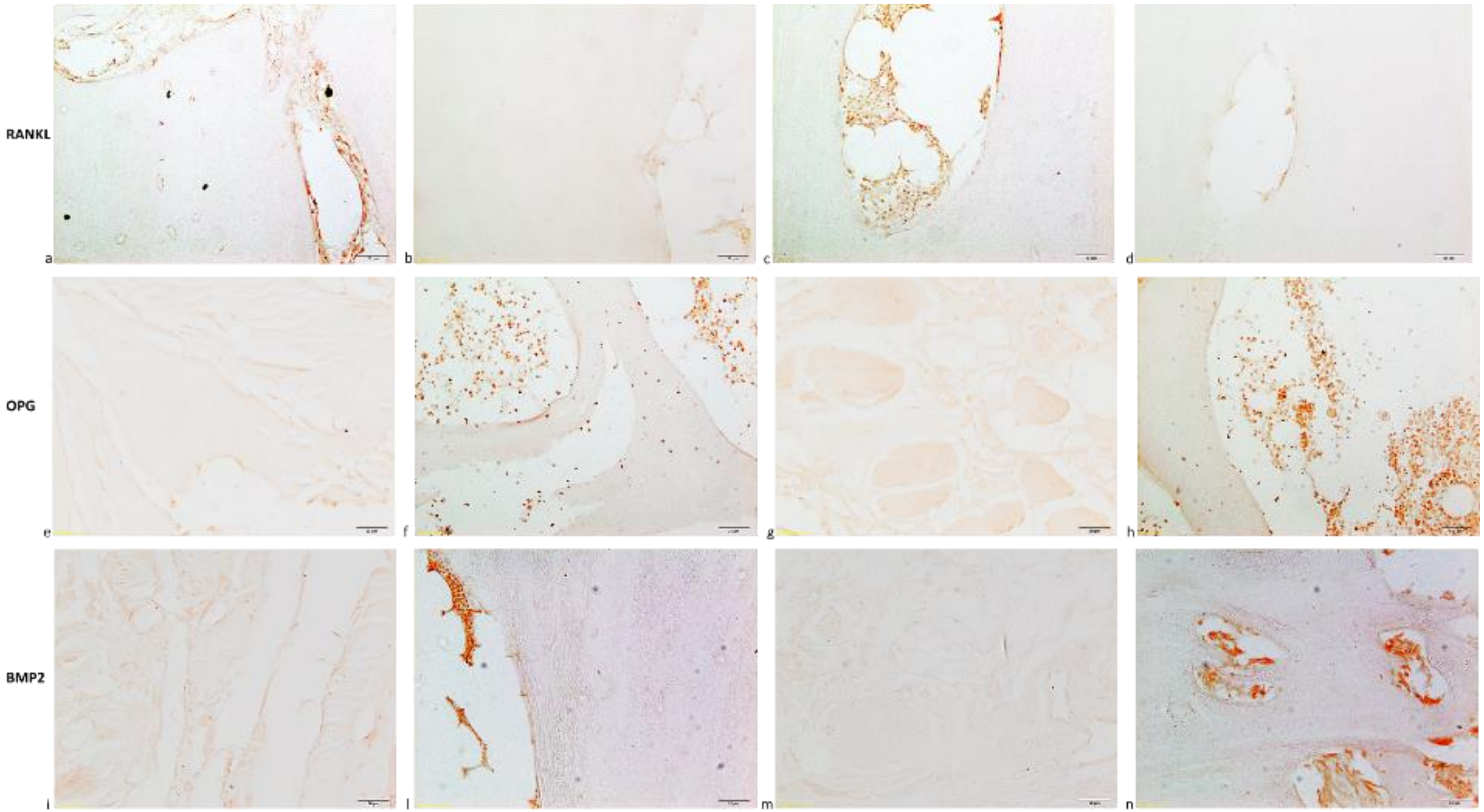

Figure S1. Representative immunoistochemical images for OP (a, e, i), SP (b, f, l), OS (c, g, m) and control (d, h, n) bone biopsies. The presence or absence of immunostaining was determined by immunohistochemistry using RANKL, OPG and BMP2 (Magnification 40x).

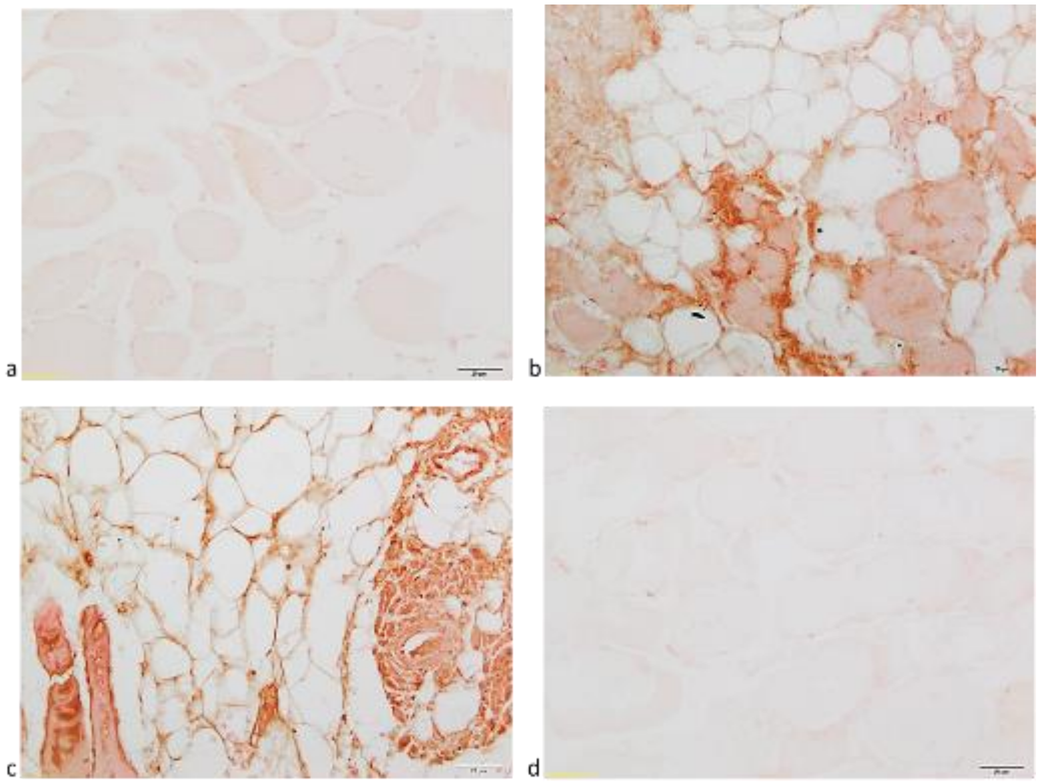

Figure S2. Representative immunoistochemical images for OP (a), SP (b), OS (c) and control (d) muscle biopsies. The presence or absence of immunostaining was determined by immunohistochemistry using TNFα (Magnification 40x).

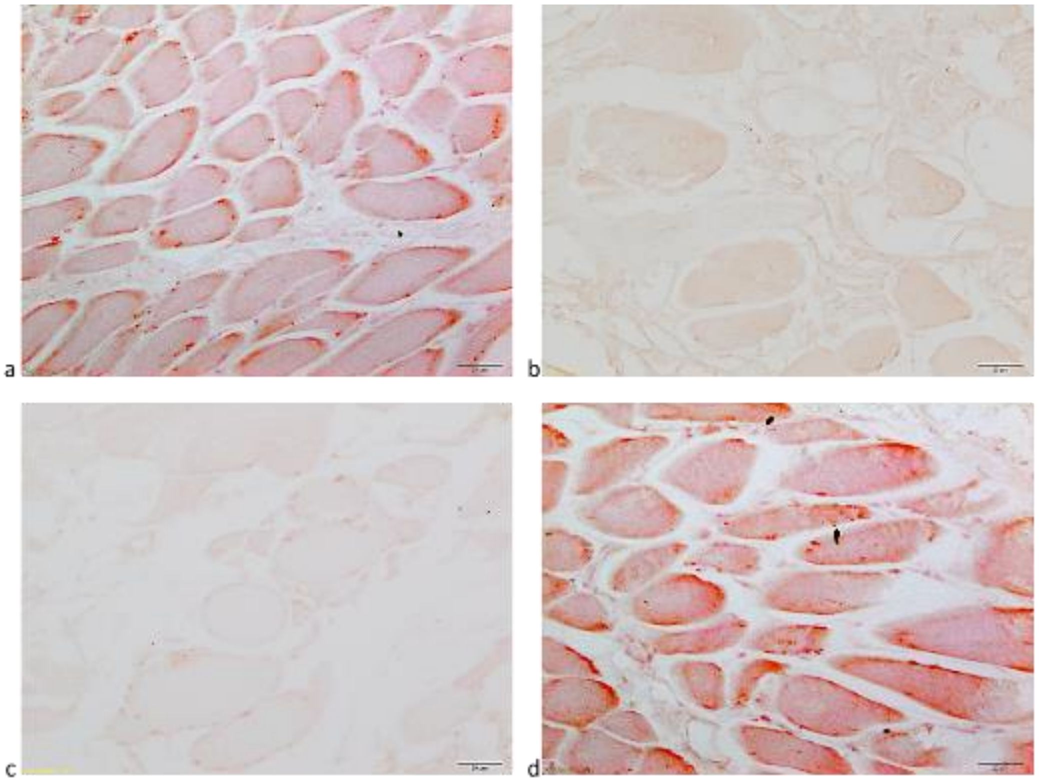

Figure S3. Representative immunoistochemical images for OP (a), SP (b), OS (c) and control (d) muscle biopsies. The presence or absence of immunostaining was determined by immunohistochemistry using COL1A1 (Magnification 40x).

2.6 Linear Discriminant Analysis (LDA)

The objective of these preliminary LDA was to identify among the blood parameters that had showing significant differences for both types of comparisons (OP, SP, OS groups vs. Control group, and among OP and SP vs. OS), those that are able to correctly classify the sample of patients into the 4 categories OP, SP, OS and Control. The blood parameters that were considered in the subsequent analyses were %Basophils, INR, aPTT, IL-15, TNF-α, FGF2, Alpha-KLOTHO, DEHA-S, and spontaneous osteoclastogenesis in termid OC counts (#OC).

Firstly, a correlation analysis was conducted among these blood parameters and the histomorphometric (BV/TV, Tb.Th, Tb.N, Tb.SP, and #Fibers) and immunohistochemical (OPG, RANKL, BMP2, TNF-a, and COL1A1) parameters, to select the blood parameters that had at least three or more significant correlations ($|r| \geq 0.65$, $p < 0.005$). Figure S4 reports the correlation plot; IL-15, FGF-2, TNFa, Alpha-KLOTHO, DEHA-S, and #OC were selected for LDA.

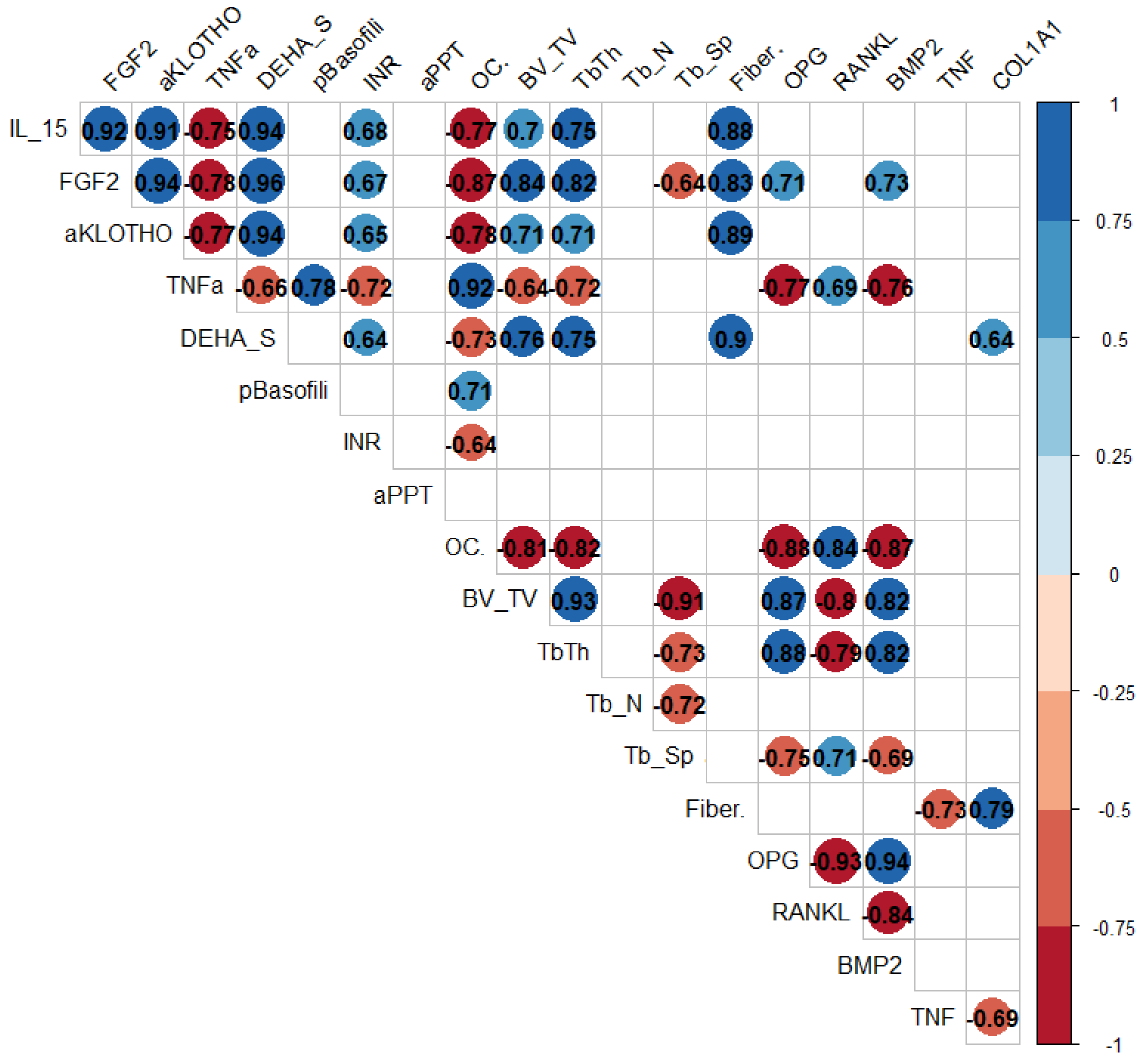

Figure S4. Correlation plot of the results of selected blood parameters and those of histomorphometric and immunohistochemical analyses. Color bar represent the variation in r-values.

Except for #OC, the other predictors did not show a univariate normal distribution and it was necessary to perform a logarithmic (IL-15, FGF2 and Alpha-KLOTHO), quadratic (TNFα) or Box-Cox (DEHA-S) transformation. None of these predictors had outliers. Having then identified multicollinearity between FGF2 and all the other predictors, between IL-15 and Alpha-KLOTHO and DEHA-S, and between #OC and TNFα, LDA was conducted on the scalar predictors in the following models by associating them with the ranking factor (Groups): (1) IL-15 + TNFα; (2) DEHA-S + TNFα; (3) #OC + IL-15; (4) #OC + Alpha-KLOTHO; and (5) #OC + DEHA-S. Table S4 reports for each model training the linear discriminant coefficients and the proportion of trace that represents the proportion of between-class variance that is explained by successive discriminant functions.

Since there were not enough patients to be able to split the dataset into 70% cases for training and 30% for testing in order to conduct cross-validation of the models, after conducting the training phase of the models on the entire dataset the testing phase was conducted using a new dataset with the same number of patients randomly generated by considering the correlation values r between the predictors of the original dataset. For each model, Table S4 summarizes the results of the testing phase of the cross-validation process by reporting the classification accuracy values and specifying the percentage of correct classification for each model and group.

Table S2.

Model		Predictors	LD1	LD2	**Actual**	**Osteopenic**	**Sarcopenic**	**Osteosarcopenic**	**Control**
					**Predicted**	**5**	**6**	**4**	**3**
**Model**		**Predictors**	**LD1**	**LD2**	**Osteopenic**	**5**	3	1	0
1	Coefficients of linear discriminants	IL-15	2.68	2.24	**Sarcopenic**	0	**3**	0	0
		TNFa	-2.19	2.27	**Osteosarcopenic**	0	0	**3**	0
	Proportion of trace (%)		97.13	2.87	**Control**	0	0	0	**3**
	Accuracy %		78.8		Classifying	100%	50%	75%	100%
**Model**		**Predictors**	**LD1**	**LD2**	**Osteopenic**	**2**	1	1	0
2	Coefficients of linear discriminants	DEHA-S	5.66	2.41	**Sarcopenic**	2	**5**	0	0
		TNFa	-1.76	2.67	**Osteosarcopenic**	1	0	**3**	0
	Proportion of trace (%)		96.98	3.02	**Control**	0	0	0	**3**
	Accuracy %		72.2		Classifying	40%	83%	75%	100%
**Model**		**Predictors**	**LD1**	**LD2**	**Osteopenic**	**2**	1	0	0
3	Coefficients of linear discriminants	IL-15	-1.52	-3.18	**Sarcopenic**	3	**3**	0	0
		#OC	4.13	-2.88	**Osteosarcopenic**	0	1	**4**	0
	Proportion of trace (%)		91.09	8.91	**Control**	0	1	0	**3**
	Accuracy %		66.7		Classifying	40%	50%	100%	100%
**Model**		**Predictors**	**LD1**	**LD2**	**Osteopenic**	**3**	1	0	0
4	Coefficients of linear discriminants	Alpha-KLOTHO	-2.07	-4.07	**Sarcopenic**	2	**4**	0	2
		#OC	3.61	-3.85	**Osteosarcopenic**	0	1	**4**	0
	Proportion of trace (%)		85.37	14.63	**Control**	0	0	0	**1**
	Accuracy %		66.7		Classifying	60%	67%	100%	33%
**Model**		**Predictors**	**LD1**	**LD2**	**Osteopenic**	**4**	2	2	0
5	Coefficients of linear discriminants	DEHA-S	5.69	3.21	**Sarcopenic**	1	**3**	0	1
		#OC	-4.21	3.33	**Osteosarcopenic**	0	0	**2**	0
	Proportion of trace (%)		95.73	4.27	**Control**	0	1	0	**2**
	Accuracy %		61.1		Classifying	80%	50%	50%	67%
